# A Deep Learning Based Approach for Grading of Diabetic Retinopathy Using Large Fundus Image Dataset

**DOI:** 10.3390/diagnostics12123084

**Published:** 2022-12-07

**Authors:** Ayesha Mehboob, Muhammad Usman Akram, Norah Saleh Alghamdi, Anum Abdul Salam

**Affiliations:** 1 Computer and Software Engineering Department, College of E&ME, National University of Sciences and Technology, Islamabad 44000, Pakistan; 2 Department of Computer Sciences, College of Computer and Information Sciences, Princess Nourah Bint Abdulrahman University, P.O. Box 84428, Riyadh 11671, Saudi Arabia

**Keywords:** diabetic retinopathy, convolution neural network (CNN), random forest tree (RFT), long short term memory (LSTM)

## Abstract

Diabetic Retinopathy affects one-third of all diabetic patients and may cause vision impairment. It has four stages of progression, i.e., mild non-proliferative, moderate non-proliferative, severe non-proliferative and proliferative Diabetic Retinopathy. The disease has no noticeable symptoms at early stages and may lead to chronic destruction, thus causing permanent blindness if not detected at an early stage. The proposed research provides deep learning frameworks for autonomous detection of Diabetic Retinopathy at an early stage using fundus images. The first framework consists of cascaded neural networks, spanned in three layers where each layer classifies data into two classes, one is the desired stage and the other output is passed to another classifier until the input image is classified as one of the stages. The second framework takes normalized, HSV and RGB fundus images as input to three Convolutional Neural Networks, and the resultant probabilistic vectors are averaged together to obtain the final output of the input image. Third framework used the Long Short Term Memory Module in CNN to emphasize the network in remembering information over a long time span. Proposed frameworks were tested and compared on the large-scale Kaggle fundus image dataset EYEPAC. The evaluations have shown that the second framework outperformed others and achieved an accuracy of 78.06% and 83.78% without and with augmentation, respectively.

## 1. Introduction

Diabetic Retinopathy (DR) is a chronic eye disease, commonly found in elderly people (age 50 or above), and can cause severe visual impairments or even blindness if not treated at an early stage [1]. The progression of DR can be categorized into four stages (as per the clinical standards), where the perceptible symptomatic appearances of the disease can only be visualized in the last stages when it becomes nearly impossible to fully recover the vision loss. DR is caused by the blood vessels rupturing due to high blood sugar levels. These leaky vessels produce fluid clots and oxygen deficiency, leading to severe visual impairments. Clinically, DR is classified into two types, i.e., proliferative and non-proliferative Diabetic Retinopathy (NPDR). NPDR is an early DR stage in which retinal lesions such as hard exudates, microaneurysms, hemorrhages, and cotton wool spots appear on the macula. If the disease is left untreated, then abnormal blood vessels from choroid intercept the retina, resulting in choroidal neovascularization. This is the severe stage of DR, dubbed as proliferative DR (PDR), which may result in complete blindness.

Apart from this, NPDR is further graded into three stages, i.e., mild, moderate, and severe NPDR, where the severe NPDR is transitioned into PDR due to the lack of proper and timely treatment [2]. Moreover, according to a recent survey, around 333 million diabetic subjects are forecasted to have a high risk of retinal abnormalities (especially DR), thus requiring proper retinal examination by 2025 [3]. At present, 387 million people are diagnosed with diabetes mellitus (DM) worldwide, with an estimated increase in count to 592 million people by 2035 [4]. Among these 387 million people, 93 million people are with positive DR [5]. The alarming figure of 28 million people out of those 93 million people have reached the vision-threatening stage. Patient vision impairment/eyesight loss count can be effectively reduced with early/timely detection of DR with a certainty of accuracy. Overall, 5–8% of people with early-stage DR can be treated with laser technology.

According to a survey conducted by WHO, improper examination or late diagnosis might leads to permanent blindness in diabetic patients. However, the progression of the disease can be reduced if diagnosed at an early stage. The intelligent self-learning and decision-making system designed to process medical images for the detection and classification of DR can be used as a decision support system to detect and stop the progression of disease at an early stage. Autonomous disease detection systems provide an efficient and timely solution with the ease of non-invasive image acquisition.

In the proposed algorithm, we have designed deep learning framework to accurately classify the detected fundus image into one of the five stages of DR. The major research contributions covered in the paper are as follows:

1. A custom lightweight CNN is proposed to handle a complex multi-class problem.
2. A new pre-processing pipeline including different color spaces for fundus images is proposed to compliment CNN architecture, which has lowered the burden from CNN architecture.
3. A new ensemble approach is presented to handle inter-class similarities in a robust manner.
4. Deep features are complimented with a random forest classifier to reduce trainable parameters.

This research article is organized as follows. Starting from Section 1, i.e., a brief introduction followed by Section 2, comprised of literature review highlighting the research gaps in this area. Section 3 explains the proposed methodology in detail, i.e., all three proposed frameworks along with the parameter details, and classifier selection. Results, comparison and dataset details are elaborated in Section 4 followed by the conclusion, i.e., Section 5. Lastly, All of the citations are referred.

## 2. Literature Review

Diabetic retinopathy is one of the most widely studied areas with respect to the development of computer-aided diagnostic systems in the last 3 decades. The developed techniques covered conventional image processing machine learning, optimization, deep learning, and now explainable models. Wang Yu, proposed a research [6] framework, which used advanced retinal image processing, deep learning, and a boosting algorithm for high-performance DR grading. The pre-processed images are passed through a convolutional neural network for the extraction of features, then the boosting tree algorithm was applied to make predictions. MK et al. [7] used a different approach that focused only on important features and exudates on retina to predict the condition of the disease, since they play a significant role in detecting the severity of disease. They used scale-invariant feature transform (SIFT) and speed-up robust features (SURF) to extract the features of exudate region. Later applied support vector machine (SVM) for the detection of DR and achieved sensitivity of 94%. P.W. et al. [8] worked on basic CNN architecture of VGG and achieved an accuracy of 99.9% using the Messidor dataset and 98% accuracy using the Kaggle dataset. They classified input data into one of three classes i.e., no DR, non-proliferative DR, and proliferative DR.

F. Alzami et al. [9] used fractal dimensions for retinopathy detection. Fractal dimensions are used to observe the vascular system of the retinal part of the eye, it not only detects the DR, but also the severity of the disease. The proposed algorithm, evaluated on the Messidor dataset using a random forest classifier, provided satisfactory results for the detection of DR, but the results were not very promising for the classification. They further concluded that other features such as univariate, multivariate, etc., can be used for testing the proposed algorithm. Another proposed algorithm [10] used inception-v3 for the feature extraction by adaptive learning the customized different classification functions of inception-v3. They used 90 retinal images for training the framework followed by testing using 48 retinal images. For classification of these deep features they used support vector machine (SVM). Results were classified into two classes only including normal and diabetic retinopathy images. D.K. Elswah et al. [11] first pre-processed the images using normalization and augmentation, followed by feature extraction using ResNet, and then performed classification on the features into one of the five classes of normal, mild, moderate, severe, and proliferative. They used Indian Diabetic Retinopathy Image Dataset (IDRiD) for their research validation and testing.

In [12], Alexander Rakhlin represented his work for Diabetic Retinopathy Detection (DRD) by designing a Deep Learning framework. He trained and tested the proposed algorithm on the Kaggle dataset, and achieved sensitivity, and specificity of 92% and 72%, respectively. The network was trained for binary classification and lacks the categorization of DR with respect to severity level. The architecture used in the project originated from famous VGG-Net family, winner of ImageNet Challenge ILSVRC-2014, designed for large-scale natural image classification. As a pre-processing step, retinal images were normalized, scaled, centered, and cropped to 540 × 540 pixels. Pratt and Coenen in [13] used a CNN approach for the diagnoses of DR using digital fundus images and also classifying its severity accurately. They developed an architecture and applied pre-processing techniques such as data augmentation for non-biased identification of the intricate features, exudates, micro-aneurysms, and hemorrhages. They used GPU to run the Architecture and tested it on 80,000 images from publicly the available Kaggle dataset. They achieved a sensitivity of 95% and an accuracy of 75% on 5000 validation images for five class classifications of DR. Ratul and Kuntal proposed a methodology in [14] using a 24-layered customized CNN architecture. In pre-processing, images are de-noised so that micro-aneurysms and hemorrhages become prominent on the retina. Images were resized, normalized, and de-noised using Non-Local Means De-noising (NLMD) and class imbalance removal by over-sampling data. Theano python was used for development and GPU for processing. The Kaggle dataset was used for training and testing. On the data set of 30,000 images, an accuracy of 95% was achieved for two-class classification and 85% accuracy for five-class classification. Zhou worked to conserve the computational resources that are invariably demanding due to deep architecture and high-resolution images [15]. They used a multistage model where the complexity of architecture and resolution of images improves at every stage. Images were labeled at each stage and labels of different stages were related. According to results on the Kaggle dataset, they received a kappa of 0.841. Romany merged two kinds of classifiers. He used a neural network for feature extraction and then performed classification on the obtained features using other algorithms [16]. Initially, image quality was improved with the help of histogram equalization. Before feature extraction, an adaptive-learning-based algorithm (Gaussian Mixture Model (GMM)) for region segmentation was performed followed by region of interest (ROI) localization using connected component-analysis. As a result, blood vessel extraction/segmentation was achieved. AlexNet DNN is used to extract features and the support-vector-machine (SVM) classifier has been for classification. Different types of features were extracted including FC7 (features of the layer before classification in CNN), linear discriminant analysis (LDA), and principle component analysis (PCA). The algorithm was evaluated on Kaggle fundus images dataset resulting in an accuracy of 97.93% using FC7 features. On the other hand, by using PCA, it showed 95.26% accuracy. Using Spatial Invariant Feature Transform (SIFT) algorithm comparative analysis showed an accuracy of 94.40%.

Guo et al. used CNN [17] for the detection of exudates in fundus images. An auxiliary loss and boosted training method was used for speedy and improved CNN training. They used U-Net, a special architecture, for exudates, and neural structure detection. They emphasized that images are classified as normal, completely ignoring the exudates (owing to the small size). During pre-processing, Contrast Limited Adaptive Histogram Equalization (CLAHE) was used on green channel only since it holds the most significant information among all planes. It enhanced the contrast between exudates and non-exudates. They proposed a boosted training method, which applied different weights to samples to vary their significance during training and used small patches as input instead of the whole images. The learned weights were tested on their own dataset and other three publicly available datasets. The obtained accuracy was 0.98, 0.96, 0.94, and 0.91, respectively. In 2022 [18], Nojood et al. proposed a deep learning framework using Inception as a baseline model to grade diabetic retinopathy using fundus images. Proposed algorithm in-cooperated Moth Optimization to enhance the algorithm efficiency. To extract ROI and lesions, histogram-based segmentation is applied followed by deep feature extraction. The algorithm yielded an accuracy of 0.999 on Messidor dataset.

Rahman et al. [19] proposed a deep learning framework to grade fundus images as mild, moderate, or severe based on the lesions and exudates. Fundus images are passed to DenseNet-169 after resizing followed by data augmentation. Extracted deep features were used to classify the image among one of the four stages of diabetic retinopathy. Training and testing of the proposed framework were performed on Kaggle APTOS 2019. The proposed model was compared with pre-trained models DenseNet-121 and ResNet-50, and resulted in the highest accuracy of 96.54%. In 2022 [20], a novel deep learning-based algorithm was proposed to accurately classify a fundus image among different stages of diabetic retinopathy and Macular Edema using order one and two 2D Fourier–Bessel series expansion-based flexible analytic wavelet transforms. Local binary patterns and variance were extracted from the selected bands to represent the statistical properties of the extracted regions using a feature vector. Various classifiers, i.e., Random Forest, K-Nearest Neighbour, and Support Vector Machines, were used to test the accuracy patterns on Messidor and IDRiD [21], resulting in an accuracy of 0.975 and 0.955, respectively. Mohamed et al. [22] devised an algorithm for diabetic retinopathy classification using DenseNet-169 as a baseline model followed by CNN to enhance the classification capability of the model. The model was trained and tested on the APTOS dataset, yielding an accuracy of 97% for binary classification, i.e., healthy or Diabetic retinopathy. The proposed framework lacks the severity-based grading of diabetic retinopathy. Novel cross-disease diagnostic CNN frameworks [23,24] were proposed for diabetic retinopathy and Macular Edema using disease-specific and disease-dependent features. IDRiD and Messidor dataset have been used to evaluate the proposed algorithms, yielding an accuracy of 92.6% and 92.9%, respectively. Wang et al. [25] proposed a cascaded 2-Phase framework. The first module of the proposed framework classifies an input fundus image as diseased or normal based on deep features. In the second phase, the diseased fundus image is further classified among four stages using a novel lesion attention module. The results of three baseline models, i.e., MobileNet, ResNet-50, and DenseNet-121, were evaluated using the DDR dataset. The highest accuracy of 80.29% was observed using DenseNet-121 Model. A CNN based hybrid model [26] was proposed to categorize a retinal input image as healthy or non-healthy based on the lesions, exudates, etc. The proposed model utilized the ResNet and DenseNet architecture to learn multi-scale features and resolve diminishing gradient problem. Model training and testing were done on a local dataset annotated by ophthalmologists, yielding an accuracy of 83.2%. Eman et al. [27] proposed an algorithm to detect various severity levels of diabetic retinopathy using retinal image dataset. The proposed framework takes a retinal image and computes PCA for each channel, followed by classification of each channel using neural network architecture. Final results were concluded based on majority voting by accumulating the results of all three channels. The algorithm yielded an accuracy of 85% when tested on DRD (Kaggle Competition dataset) with retinal images belonging to all severity levels.

An ensemble-based machine learning algorithm proposed in 2021 [28] incorporated three different classifiers, i.e., Random Forest, SVM, and Neural Networks, followed by a meta classifier to reach a decision. Using an ensemble-based approach, robustness and performance have been enhanced. The proposed algorithm was tested on the Messidor dataset and yielded an accuracy of 0.75. Another ensemble-based algorithm was proposed for diabetic retinopathy screening in 2021 [29]. A two staged classifier has been proposed, where the first stage was comprised of outputs from six classifiers, i.e., SVM, KNN, Multilayer perceptron, Naive Bayes, Decision Trees and Logistic Regression, followed by the second stage, i.e., a neural network, which utilized the output from classifiers to reach the final decision. The proposed algorithm resulted in a test accuracy 76.40% when tested on the Messidor dataset. Another ensemble-based deep neural network architecture was proposed using ResNet in 2020 [30]. The proposed algorithm utilized four ResNets to binary classify among the five classes of DR, i.e., normal vs. mild DR, normal vs. moderate DR, normal vs. severe DR, and normal vs. proliferative DR. The results from each classifier from stage-1 were then processed by AdaBoost classifier in stage-2 to reach the final classification results. The algorithm was evaluated on the Kaggle dataset APTOS 3662 retinal images, resulting in an accuracy of 61.9%.

In a comprehensive review of state-of-the-art algorithms, we have concluded various research gaps in diabetic retinopathy autonomous detection systems. The majority of the research algorithms yielding good results are catering to the problem of binary classification, i.e., healthy vs. non-healthy image or a three-class classification, and thus might result in the incorrect classification of severe cases, which might lead to permanent loss of sight. Keeping this in view, because of data distortions, it is difficult to deal with all five classes at the same time, and we tried to resolve the matter of class imbalance with our different frameworks. Finding the appropriate set of pre-processing steps to extract images that provide the best results possible is also one of the milestones achieved in the research. Focusing on the features of interest and enhancing the main features required for the targeted problem can help improve the results.

## 3. Methodology

**Figure 1:**
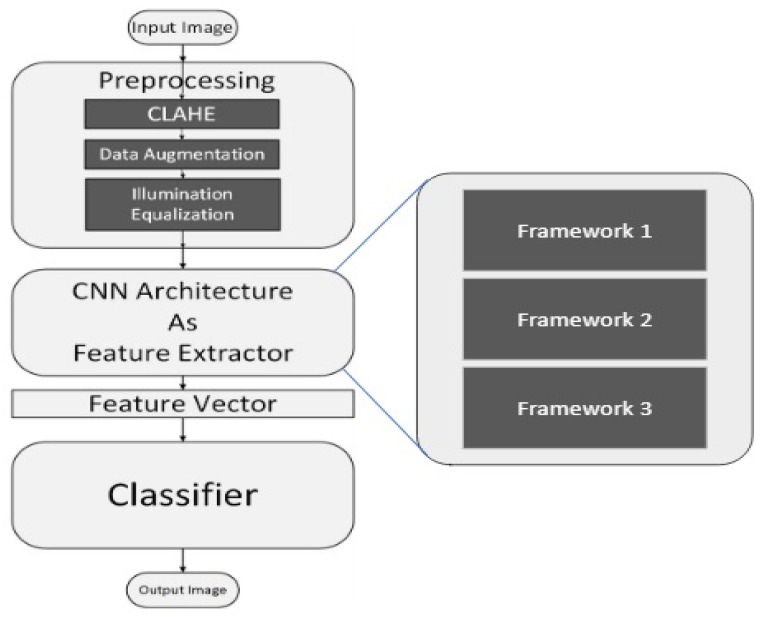
Block diagram of the proposed algorithm.

The proposed architecture is comprised of three phases, i.e., image pre-processing, feature extraction followed by classification. We have used the Kaggle dataset to train/test the proposed frameworks. The flow chart diagram of the proposed work is given in Figure 1. To extract deep features, we trained Deep Convolutional Networks. Heat Maps extracted from the proposed framework highlight the presence of any exudates, microaneurysms, hemorrhages, cotton wool spots, or new build vessels; which indicate the extraction of features from the affected region, thus yielding high accuracy. The deep CNN is capable of taking unknown images as input and extracting problem-specific features, thereby generating an appropriate response. CNN, improving its feature extraction during every iteration of backpropagation, converges closer to the optimum solution for the specific problem under consideration. Based on the decision-making of CNN, it is decided to update the parameter weights in case of false positives or to retain the parameter weights in case of true positives. Invariably, the working capabilities of CNN are dependent on the quality and nature of images; therefore, we resorted to pre-processing instead of feeding the system with raw images. During the pre-processing, we have enhanced the minute details in order to make exudates and micro-aneurysms more prominent. We have further carried out light equalization as the images were taken in light conditions of varying intensities. To conserve the computational resources, images were resized and the background was removed. The pre-processed images were then forwarded to our deep CNN architecture, which extracted the best-defined features to signify the objects of our interest within the images. These collected features are fed to a classifier for DR classification into normal, mild, moderate, severe, or proliferative. As highlighted in Figure 1, we have proposed three different CNN-based frameworks to categorize an image among five classes. Lastly, the accuracy of all the frameworks has been compared to analyze the performance. Below are the details of each of the CNN-based proposed frameworks.

1. Framework 1 used a basic CNN Architecture for feature extraction; these deep features are defined to represent our objects of interest within the images and are classified using a conventional Random forest classifier [31] (showed the best accuracy among many other classifiers), and designed a cascaded architecture. In this architecture, multiple cascaded layers are used to classify an image into one of the five classes.
2. Framework 2 performed multiple pre-processing techniques on dataset to compare their results for five class classifications and created an improved ensemble result using them. We applied CNN Architecture of Framework 1 on RGB, HSV and normalized images, categorized as CNN-1, CNN-2, and CNN-3, respectively.
3. Framework 3 classified images by creating a series of patches (9 patches from each image), and classification was done using LSTM-based CNN Architecture.

### 3.1. Stage 1: Pre-Processing

**Figure 2:**
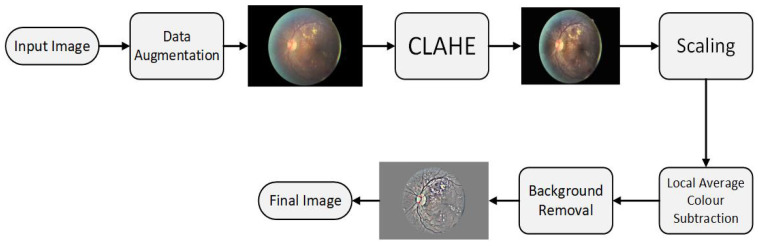
Pre-processing phase of input image.

**Figure 3:**
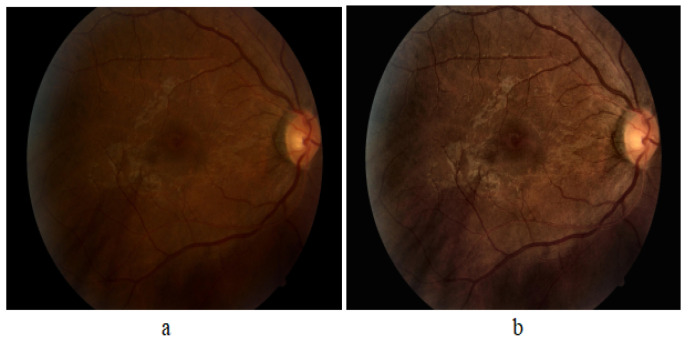
Effect of applying CLAHE; (**a**) original; and (**b**) CLAHE processed.

The first stage of the proposed architecture is the pre-processing phase. The input images are not standardized as they have an un-wanted black background, noise, different aspect ratios, varying light conditions, different color averages, and imbalanced classes. An algorithm shown in Figure 2 has been designed to pre-process the raw images and make it optimal. The steps included:

1. **Data Augmentation.** In order to solve the issue of class imbalance, we applied data augmentation to the dataset. A huge class imbalance existed among the dataset. With class 0 representing more than 50% of the dataset, portions of the dataset represented by the classes in terms of percentage are shown in Table 1. There was a great possibility of over-fitting. As the system will be seeing normal class images way more than any of the other 4 classes, ultimately the trained parameters will learn to lean towards normal class. We over-sampled the images of less dominant classes, using the regeneration of new images from the existing ones by changing a few details in them. We have applied the following augmentations (transformations) during our research:
  (a) Flipping to 90 degrees.
  (b) Rotating images by [0, 180] degrees with a step size of 15 degrees.
  (c) Translating (on scale x and y dimensions).
  To up-sample Class 1 and Class 2 samples, the flip transformation has been applied. Since classes 3 and 4 differ from the rest of the classes by a large percentage, therefore both of the classes were up-sampled by applying all of the transformation steps, i.e., flip, rotation, and translation to reduce the skewed output of the trained model. The classwise data distribution before and after augmentation is shown in Table 1.
2. **CLAHE.** We used Contrast Limited Adaptive Histogram Equalization (CLAHE) technique for the enhancement of an image. It performs a very clear and detailed contrast improvement in an image by equalization of lighting effects. The enhancement results are remarkable even for low-contrast images (underwater images), evident in Figure 3. It can be seen that applying CLAHE on retinal images has enhanced the visibility of minute details. The working of CLAHE is as follows:
  (a) Step 1 Division of image into small equal-sized partitions.
  (b) Step 2 Performing histogram equalization on every partition.
  (c) Step 3 Limiting the amplification by clipping the histogram at some specific value.
3. **Re-scaling.** We have then re-scaled the images by standardizing the size of the optic region to have a common radius (300 pixels or 500 pixels).
4. **Background Removal, Colour Normalization andGray Scale Mapping.** We then removed the background, keeping a specific radius to focus on ROI, subtracted the local average color, thereby suppressing the unwanted details, and mapped the local average to 50% grayscale image.
5. **Resizing.** Finally, we resized the image to 256 × 256 according to the requirement of CNN.

### 3.2. Stage 2: CNN Architecture for Deep Feature Extraction

**Figure 4:**
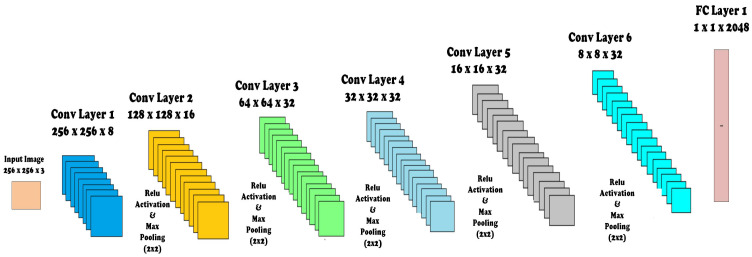
Custom design lightweight CNN architecture.

In order to improve the accuracy, we may add architectural layers to a level, after which there is a drop in accuracy due to inherent factors like over-fitting, etc. The CNN proposed in our research is comprised of layers, namely; input map, convolution layer, activation layer, and max pooling layer. The parameters of CNN, depicting the layer, number of feature maps in each layer (Activation Shape), number of features in each layer (Activation size), and weights (parameters to be trained) are shown in Table 2. The architectural diagram for custom CNN is also shown in Figure 4.

Detailed architecture of the layers are as follows:

1. **Covolution.** This layer has a set of filters (kernel), as our first layer has eight filters. Each filter should have the same depth as the depth of input, since our first layer input image is RGB with depth 3, so we have eight filters of depth 3. The input image becomes convolved with each filter and forms eight feature maps on each convolution layer. Comprising filters (kernel), feature maps are created for the input image based on the number of filters, e.g., if the first layer has 8× filters, this layer will produce 8× feature maps as a result of input image convolution with each filter. For an image of size k∗l and filter of size m∗n, each feature map will have size (with 2 × 2 zero padding and stride = 1):
  (1) [(k−m+1)+2]∗[(l−n+1)+2]
  For 8× filters of size m∗n, the trainable parameters to collect features from image will be:
  (2) (m∗n∗8)+1
  where 1 is added for bias.
2. **MaxPooling Layer.** Max pooling is used to reduce the size (down sample) of the coming feature maps intelligently so that most of the information remains intact. With the reduced dimensions, parameters are also reduced as we move forward in the network. Therefore, it reduces the chances of over-fitting and becomes an efficient system with less computational cost. We used a kernel of size 2 × 2 in each max-pooling layer, and it selects the maximum number from 2 × 2 frame and the frame moves with a 2 × 2 stride. This layer has no parameters that are to be trained.
3. **Activation Layer.** In order to make an effective system for complicated problem solving, an activation function has been added. We used a rectified linear unit (ReLU) after every convolutional and Fully Connected layer since it resulted in the highest accuracy among other activation functions and prevented the vanishing gradient problem effectively. We have designed ReLU within the convolution layer in our architecture, and it has improved the accuracy of classification.
4. **Dropout Layer.** We have avoided the over-fitting by using the dropout layer, as it prevents the network dependence on single node. Probability is assigned to each node, which decides its value.
5. **Fully Connected (FC) Layer.** After passing through many convolutions, activation and max-pooling layers, the most important feature is concentrated. Feature maps of the last layer are then flattened, enabling them to be fed into the FC layer, where every feature value is connected to every neuron. We have used an FC layer consisting of 2048× neurons. The number of parameters to be trained for this layer are represented as:
  (3) Numberoffeatures∗Numberofneurons
6. **Feature Vector.** After this FC layer, we collected these 2048× features of every image as labelled feature vectors for subsequent training of our classifier.

### 3.3. Stage 3: Classification Using Classifiers

We have tested our system with various classifiers, i.e., Naive Bayes Logistic Regression, Simple Logistic, SVM, 3NN, Bagging and Random Forest. Among all, Random Forest Tree (RFT) has proven to be most suited classifier for grading of DR. As suggested by its name, RFT is structured on the idea of decision trees in which a decision is made at every node for an unknown problem. The nature of questions in the unknown problem should be discriminating among various classes. At every stage, data is traversed towards its destined end-node (related class) based on the decisions.

### 3.4. Framework 1: Cascaded Classifier

**Figure 5:**
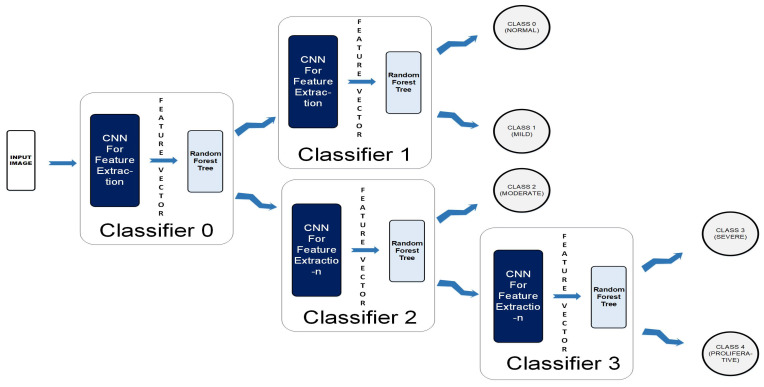
Framework 1-cascaded classifier architecture.

Instead of designing one Classifier of five classes, we have designed a Cascaded Classifier using four binary classifiers, since cascaded classifiers are computationally less expensive and require less training time [32,33]. Our Cascaded Classifier Network (CCN) is a novel deep learning architecture comprised of multiple CNNs merged with classifiers, each predicting a specific feature-set to further divide the data. The division process continues until we obtain leaf nodes equal to the number of classes. Our cascaded classifier is shown in Figure 5. This design allowed us to train all the neural networks simultaneously. We were able to reduce the possibility of error and over-fitting due to the binary nature of the proposed classifier.

1. A coloured fundus image (256 × 256 × 3) is fed to the CCN as input.
2. The output map is sequentially forwarded from the previous one to the next four classifiers within the CCN.
3. The new test color fundus image moves node-wise and ultimately reaches one of the five leaf nodes (terminal nodes) for final decision-making.
4. Finally, it can be classified among one the five classes: Normal, Mild, Moderate, Severe, and Proliferative.

**Cascaded Classifier Architecture**

1. **Level 0.** The unknown test image is partially classified at the first stage. In Classifier 0, the image goes to class 0, representing Normal and Mild Classes, or Class1, which characterizes Moderate, Severe, and Proliferative stages of DR.
2. **Level 1.** At level-1, Classifier-1 further classifies class-0 from level-1 by labelling them into Normal (Label0) or Mild (Label1).
3. **Level 2.** Using Classifier-2, class-1 can further be divided into two classes where class-0 is Label 2 (Moderate), and class 1 is Label 3 (Severe) and Label4 (Proliferative). At this level, we have acquired end nodes for the classes Normal, Mild and Moderate; however, Class 1 output from Classifier 2 needs further classification.
4. **Level 3.** During this stage, Class 1 output from Classifier 2 is further segregated and labeled as Severe (Label3) or Proliferative (Label4).
5. **Level 4.** Finally, we were able to acquire five separate classes as leaf nodes with subsequent labels as Label 0 (Normal), Label 1 (Mild), Label 2 (Moderate), Label 3 (Severe) and Label 4 (Proliferative). In our design, by making use of the simultaneous training technique, we were able to save the time originally required in case of independent classifier training.

### 3.5. FrameWork 2: Ensembled System Design

In the ensembled system, we combined different systems to get our final results. We used our CNN Architecture for the classification of three different types of pre-processed datasets, i.e., Normalized images, RGB images, and HSV images, categorized as CNN-1, CNN-2, and CNN-3, respectively.

1. **Normalized (CNN-1):** We Pre-processed Images in order to suppress unwanted information from images. This dataset is pre-processed with the same algorithm as we used in Framework 1.
2. **RGB (CNN-2):** It is additive colour representation of images. Every pixel in the image represents the saved information in the terms of color. The intensity value of the three primary colors, red, green, and blue, combined shows one color. The name RGB is also derived from the initials of the three primary colours.
3. **HSV (CNN-3):** It is a representation of an image in terms of hue, saturation and value, and the three channels represent each of these. Hue is an angle on a colored spherical surface; it gives color. Saturation is the measure of how light or dark that color is; it is a point on the radius at the angle represented by hue. Value is a measure of brightness or intensity of color; it works simultaneously with saturation.

**Figure 6:**
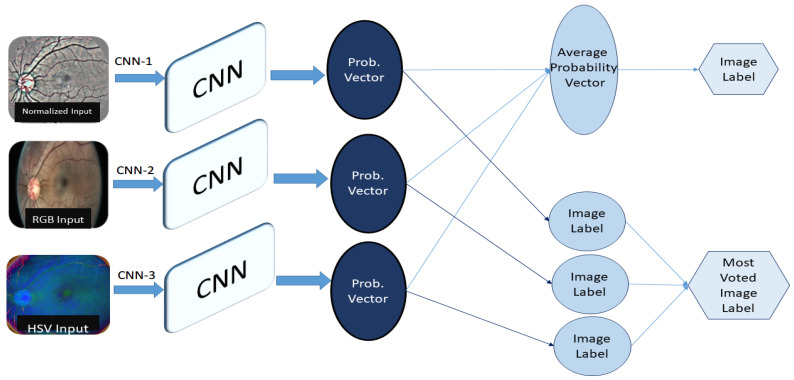
Framework 2-Ensembled System Design.

Separate results of these three different systems are observed and we also observed ensemble results in two different ways for improved accuracy as shown in Figure 6.

1. Most Voted results from three CNN classifiers were collected.
2. Average Probability vector from probability vectors of three CNNs was used to collect results.

### 3.6. Framework 3: LSTM CNN

**Figure 7:**
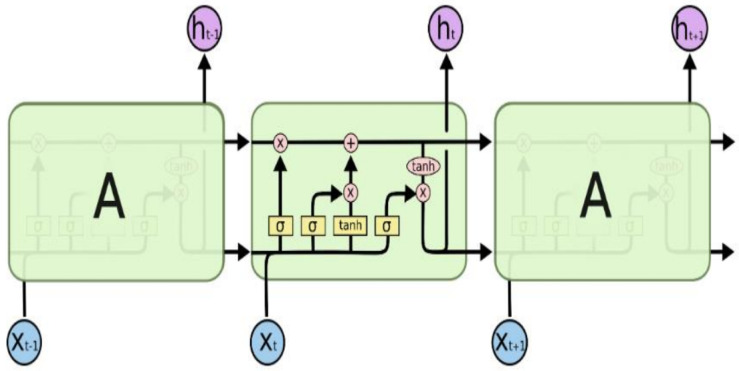
Framework 3-LSTM working [34].

Long Short Term Memory networks, usually just called LSTM, share a special kind of RNN, capable of learning long-term dependencies. LSTMs are explicitly designed to avoid the long-term dependency problem. Remembering information for long periods of time is practically their default behavior, not something they struggle to learn. The learning mechanism of LSTM is shown in Figure 7, and these working steps of an LSTM architecture are as follows:

1. The first step in our LSTM [34] is to decide what information we are going to throw away from the cell state. This decision is made by a sigmoid layer called the forget gate layer. It looks at $h_{t-1}$ and $x_{t}$, and outputs a number between 0 and 1 for each number in the cell state $C_{t-1}$. A ‘1’ represents completely keep this, while a ‘0’ represents completely get rid of this.
2. The next step is to decide what new information we are going to store in the cell state. It has two parts.
  - First, a sigmoid layer, called the input gate layer, decides which values we will update.
  - Next, a tanh layer creates a vector of new candidate values, $C_{t}$, that could be added to the state. In the next step, we will combine these two to create an update to the state.
3. It’s now time to update the old cell state, $C_{t-1}$, into the new cell state $C_{t}$. The previous steps have already reached a decision; we just need to propagate it. We multiply the old state by $f_{t}$, forgetting the things we decided to forget earlier. Then, we add $C_{t}$ to it. This is the new candidate value, scaled by how much we decided to update each state value.

Finally, we need to decide what we are propagating to the output. This output will be based on our cell state, but will be a filtered version. First, we run a sigmoid layer, which decides what parts of the cell state we are passing to the output. Then, we put the cell state through tanh (to push the values to be between −1 and 1) and multiply it by the output of the sigmoid gate, so that we only output the parts we decided to propagate.

## 4. Results

Prior to ascertaining the efficacy and accuracy of any deep learning framework, it requires extensive training using a valid and diverse dataset. In our system design, we have performed training and testing using Kaggle’s Diabetic retinopathy dataset. We have evaluated the proposed architecture using accuracy, sensitivity, and specificity as evaluation metrics.

### 4.1. Kaggle Dataset

The Kaggle dataset [35] (provided by EyePACS), has for diabetic retinopathy, a collection of fundus eye images, publicly available for a comparison of detecting Diabetic Retinopathy using Neural Networks, and has been used for training, testing, and validation of the proposed algorithm. It consists of a total of 88,702 images of which 35,126 are training images and 53,576 are testing images. These high-resolution images consisting of left and right eye images are taken under different conditions. Clinicians have rated the images with presence (detection) and severity (grading) of DR on a scale of 0 to 4, where 0 represents Normal or No DR Disease, 1 signifies Mild disease, 2 specifies Moderate disease, 3 represents Severe disease and finally, 4 signifies Proliferative DR disease. In this dataset about 73% of the total images belongs to class 0 (Normal or No DR Disease). Whereas, 708 images are labeled as Proliferative DR, i.e., the highest stage on the severity level, which might lead to blindness if left untreated. In order to resolve the class imbalance problem in the dataset, we have applied augmentation on the classes having the least contribution in training the model. Table 1 elaborates the class-wise effect on training dataset size before and after augmentation, resulting in an increase of training data samples from 35,126 to 96,213. Classes with fewer training samples are up-sampled by applying Flip, Rotation, and Translation along the x and y planes.

### 4.2. Framework 1: Cascaded Design

1. After obtaining feature vectors from the trained neural network, we have performed testing using more than one classifier and have resorted to Random Forest Tree (RFT) based on its accuracy. The performance metrics for different classifiers are given in Table 3. The highest accuracy of 74% has been achieved using Random Forest Classifier.
2. After the confirmation of the best classifier for the problem under consideration, we used it in our cascaded design as depicted in Figure 5. Table 4 shows how each stage is divided into various sub-classes, i.e., class 0 vs. class 1. Accuracy for each level through the Cascaded Classifier along with confusion matrices are shown in Table 5. Using this architecture, we overcame the problem of class imbalance in the dataset. Stage 3 shows the lowest accuracy as the two classes were close to each other and intra-class variance is quite low. Whereas, stage 1 shows the highest accuracy since there is a significant difference between Normal and Mild PDR. This architecture helped make class-wise observations and highlighted the accuracy achieved by grouping the classes.
3. By making use of our detection and classification system, we were able to achieve an average accuracy of 78.5%.

### 4.3. Framework 2: Ensemble System

In Ensemble-based architecture, the highest accuracy has been achieved using normalized input retinal images. Table 6, Table 7 and Table 8 elaborate the confusion matrices of CNN-1, CNN-2 and CNN-3, respectively. In CNN1, a large number of false positives for the moderate class are mapped on severe and PDR because of high textural similarities. Similarly, a high false positive mapping has been observed among severe and PDR for each other. A detailed confusion matrix for CNN 1 using color Normalized images as input for a five-class classification Confusion matrix is shown in Table 6 with an accuracy of 81.68%, the sensitivity of 78.36% and specificity of 95.08%.

CNN2, trained using RGB images, mapped a number of false positives of Normal class towards Mild and Moderate. False mapping among Severe and Proliferative DR persisted in this model. CNN 2 achieved an accuracy of 79.2%, the sensitivity of 80.3%, and specificity of 94.4%. An elaborated confusion matrix for CNN2 has been presented in Table 7.

In CNN3, using HSV images, a high rate of false positive mapping has been observed for the moderate class. Similarly, the complexity among the classification of severe PDR existed in the model. The model achieved an accuracy of 75.67%, sensitivity of 80.12%, and specificity of 94.19%. However, averaging the results from all CNN models depicted in Table 8 helped reduce the false positives of all classes, therefore increasing the one vs. all class accuracy. Furthermore, applying average pooling on the results from each classifier improved the class accuracy, specifically among severe and PDR cases, thus enhancing the performance of the proposed architecture.

The confusion Matrix along with accuracy after infusing the results in two different ways from prior three CNN are shown in the following tables. Label collected using Average Probability vector showed comparatively improved results with accuracy of 83.78%, the sensitivity of 82.24%, and specificity of 95.52%. The most voted label collected from three CNN labels showed an accuracy of 82.79%, sensitivity of 78.55%, and specificity of 97.92%. Table 9 and Table 10 show the class-wise confusion matrix for each of the infusions, respectively.

### 4.4. Framework 3: LSTM-CNN Design

Architecture comprised of LSTM-CNN yielded a high-class accuracy, with a low tendency towards forgetting features, therefore, reduced the fall positive rate and catered the discrimination between PDR and severe cases more accurately as compared to cascaded architecture. A confusion matrix to give a visualization of correctly classified images using LSTMCNN is shown in Table 11 with an accuracy of 80.31%, sensitivity of 82.24% and specificity of 94.46%.

### 4.5. Comparison of Results

We have performed a two-level comparative analysis. In the first, we compared all the proposed frameworks to show the performance of each proposed framework. After that, the best framework is further compared with existing techniques on the same data set.

#### 4.5.1. Comparison between Proposed Frameworks

**Figure 8:**
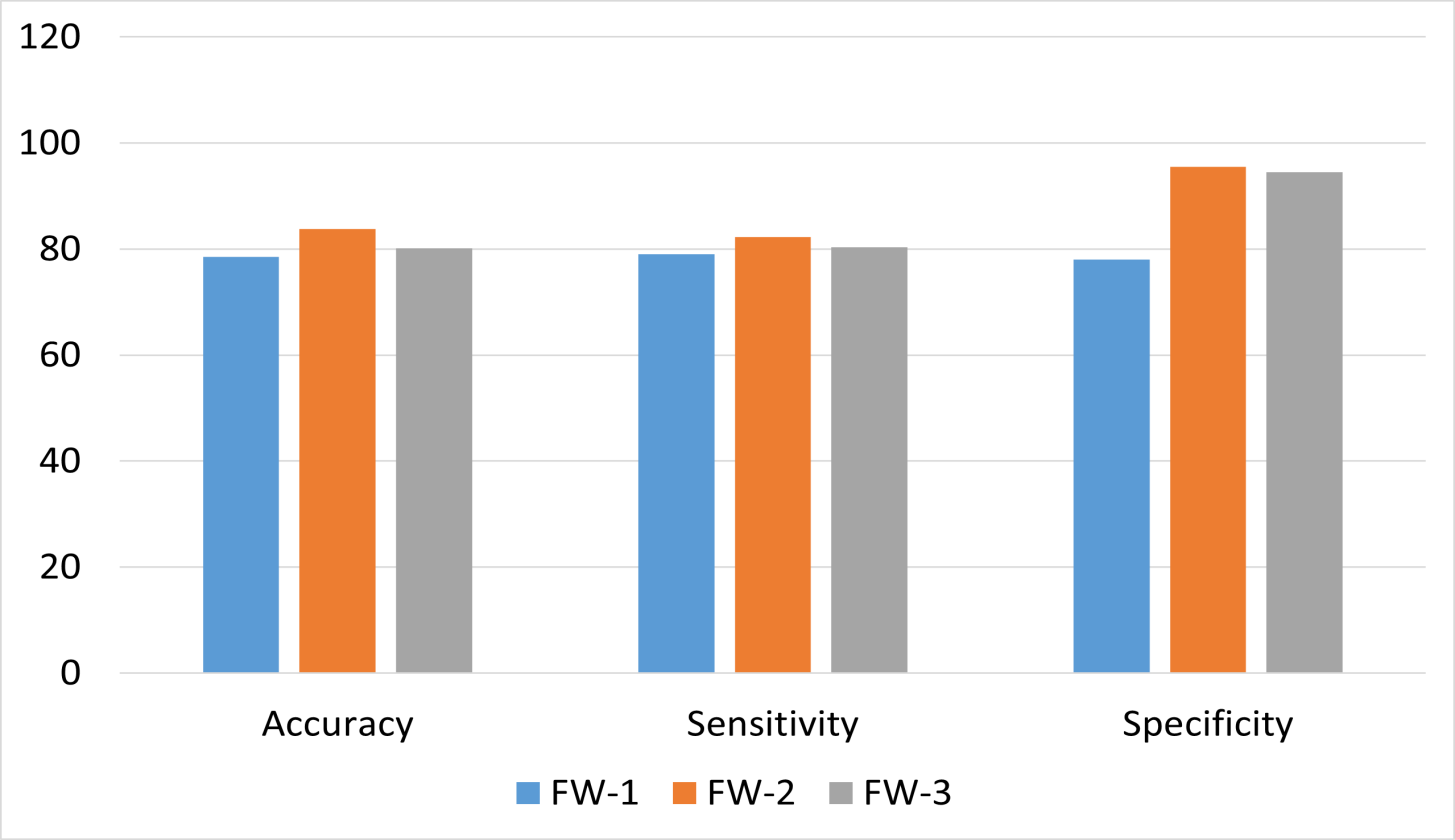
Performance comparison of all three proposed frameworks using augmented data.

**Figure 9:**
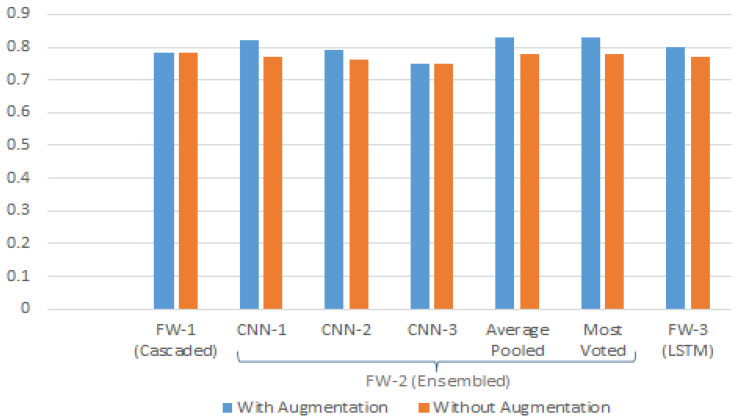
Performance comparison of proposed frameworks against augmented vs. non-augmented training dataset.

The results of our three Frameworks in accordance with their respective accuracy, sensitivity, and specificity are shown in Figure 8. It is evident from the figure that the ensemble-based system yields the highest overall accuracy using average pooling, since it catered the false negatives more accurately by average pooling the results from each of the trained CNNs, i.e., CNN-1, CNN-2 and CNN-3. Therefore, by correlating results from all three CNNs, there remains a lower chance of misclassification for severe cases of DR. Moreover, as evident from Figure 9, training the proposed architecture using the augmented dataset has significantly increased the overall accuracy of each framework, since it has added more class-wise samples of less-skewed classes, thus enhancing the class-wise true positives and true negatives for each class, resulting in an increase of overall accuracy.

#### 4.5.2. Class-Wise Comparison between Frameworks

Table 12 shows the accuracy achieved by each class of the proposed framework. According to the table, Framework 2 shows the highest accuracy for most of the classes except for the PDR. Whereas, Framework 3 shows the highest accuracy for PDR class. Framework 1 showed a low degree of accuracy among the last two severity categorizations since in cascaded architecture, the last phase of the architecture is discriminating between these two as class 0 and class 1. Since the disease landmarks for both stages are similar, the Model can find it harder to discriminate between two of them more accurately. Moreover, training samples of both classes were up-sampled using data augmentation, thus resulting the model in difficult to categorize between two classes. In framework 2, i.e., ensembled-based architecture, most voted classification leads to more accurate results rather than average pooling. This depicts the fact that averaging results from various classifiers might not conclude the results more accurately in ensemble-based architecture. Figure 9 depicts the results from each framework using augmentation-based training data vs. no augmentation-based training data. Graph clearly illustrates that ensemble-based architecture using average pooling stands out amongst all of the frameworks using augmented training data with an overall accuracy of 84%.

#### 4.5.3. Comparison with Existing Techniques

Table 13 shows the comparison between the results of proposed and previously published related work. Compared to the previous work, our system showed promising results. F. Mansour Romany [16] used deep CNN to separate data into two classes for normal and diseased with an accuracy of 97.93%, but our system is working on five class divisions and providing good class-wise accuracy. The results show that the proposed technique has outperformed existing ones in terms of sensitivity value. It also shows competitive accuracy even with a lighter CNN architecture. Moreover, among ensemble-based architecture, our proposed framework yields the highest accuracy using average pooling, when trained on an augmented dataset. Categorization of PDR and severe DR is challenging among all classes, since they hold huge similarities in terms of disease landmarks. The proposed model categorizes these two categories with relatively better accuracy.

## 5. Conclusions

The proposed algorithm categorizes a fundus image into one of the five classes of Diabetic Retinopathy. Among the proposed frameworks, an ensemble-based classification algorithm resulted in the highest accuracy. Following the given system of pre-processing and architecture design, we achieved significantly good accuracy. The result shows that the cascaded classifier of the convolution neural network and the random forest is a promising tool for the automatic Five Class Classification of Diabetic retinopathy in optimal resources. Using the cascaded system, training becomes less time-consuming, as training can be done in parallel. A good pre-processing algorithm for a challenging conditioned dataset enhances the quality measures. Moreover, apart from DR detection from retinal image, grading DR is equally important to enhance the accuracy of autonomous systems and screen out severe cases. The proposed algorithm provides high class-based accuracy for severe DR cases and PDR. To enhance the robustness of the proposed framework, testing is done on a large publicly available dataset with data augmentation for classes with relatively less samples. Clinical benefits of the system are autonomous grading of PDR using fundus images. The system could be beneficial in rural areas to achieve e-clinicians, in order to screen severe cases and recommend them to doctors for detailed examination. 

## Figures and Tables

**Table 1:** Distribution of classes in Kaggle dataset.

Label	Grade	Percentage	Train Images	Test Images	Train Images Augmented
**0**	Normal	73%	25,642	39,110	25,642
**1**	Mild	6.96%	2445	3729	22,005
**2**	Moderate	15.57%	5469	8342	21,876
**3**	Severe	2.46%	864	1318	14,688
**4**	Proliferative	2.01%	706	1077	12,002

**Table 2:** Hyper parameters of the CNN.

Layer	Activation Shape	Activation Size	Parameter
Input	256 × 256 × 3	0	0
Conv 1	256 × 256 × 8	524,288	80
Conv 2	128 × 128 × 16	262,144	160
Conv 3	64 × 64 × 32	131,072	320
Conv 4	32 × 32 × 32	32,768	320
Conv 5	16 × 16 × 32	8192	320
Conv 6	8 × 8 × 32	2048	320
FC 1	2048 × 1	2048	4,194,304

**Table 3:** Performance comparison between classifiers tested for cascaded classifier.

Classifier	Accuracy	Sensitivity	Specificity
Naive Bayse	62%	78%	44%
Logistic Regression	69%	85%	50%
Simple Logistic	70%	93%	37%
SVM	72%	93%	42%
3NN	66%	88%	36%
Bagging	70%	93%	36%
Random Forest	74%	94%	31%

**Table 4:** Class divisions (0–1) for each stage of Cascaded Classifier.

	Stage 0	Stage 1	Stage 2	Stage 3
Class 0	Normal and Mild	Normal	Moderate	severe
Class 1	Severe, Moderate and PDR	Mild	severe and PDR	PDR

**Table 5:** Confusion Matrix for each Stage in CNN. (a) Stage 0-Normal, Mild and Severe, Moderate and PDR: Accuracy 81.9%; (b) Stage 1-Normal and Mild: Accuracy 86.3%; (c) Stage 2-Moderate, Severe and PDR: Accuracy 76.2%; (d) Stage 3-Severe, PDR: Accuracy 69.6%. Average accuracy is 78.5%.

a	Class 0	Class 1	b	Class 0	Class 1
Class 0	34,752	8087	Class 0	34,107	5003
Class 1	1605	9132	Class 1	853	2876
**c**	**Class 0**	**Class 1**	**d**	**Class 0**	**Class 1**
Class 0	6389	1953	Class 0	807	511
Class 1	592	1803	Class 1	215	862

**Table 6:** Confusion matrix For CNN 1 (Normalized Images), Accuracy = 81.68%.

	Normal	Mild	Moderate	Severe	PDR
**Normal**	33,048	4219	1472	203	168
**Mild**	937	2730	52	8	2
**Moderate**	27	109	6048	1395	763
**Severe**	14	23	77	1075	129
**PDR**	2	15	73	125	862

**Table 7:** Confusion matrix For CNN 2 (RGB Images), Accuracy = 79.2%.

	Normal	Mild	Moderate	Severe	PDR
**Normal**	31,359	5012	2132	297	310
**Mild**	1032	2627	47	11	12
**Moderate**	21	97	6361	1032	831
**Severe**	4	8	18	1262	26
**Proliferative**	4	21	67	134	851

**Table 8:** Confusion matrix for CNN 3 (HSV Images), Accuracy = 75.67%.

	Normal	Mild	Moderate	Severe	PDR
**Normal**	29,217	7231	2061	317	284
**Mild**	493	3153	63	14	6
**Moderate**	37	132	6178	1142	853
**Severe**	11	22	173	1055	57
**PDR**	7	15	32	83	940

**Table 9:** Confusion matrix For averaged probability results of ensemble architecture, accuracy = 83.78%.

	Normal	Mild	Moderate	Severe	PDR
**Normal**	33,709	4103	973	152	173
**Mild**	975	2687	51	9	7
**Moderate**	21	97	6361	1032	831
**Severe**	4	7	24	1246	37
**PDR**	6	27	63	96	885

**Table 10:** Confusion matrix for most voted results of ensemble architecture, accuracy = 82.79%.

	Normal	Mild	Moderate	Severe	PDR
**Normal**	33,404	4375	952	165	214
**Mild**	1015	2640	52	10	12
**Moderate**	19	103	6221	1172	827
**Severe**	5	7	24	1243	39
**PDR**	3	20	71	137	846

**Table 11:** Confusion matrix of LSTM-CNN, accuracy = 80.15%.

	Normal	Mild	Moderate	Severe	PDR
**Normal**	31,852	5872	1036	153	197
**Mild**	1262	2382	43	27	15
**Moderate**	15	132	6565	1007	623
**Severe**	3	10	17	1261	27
**PDR**	4	21	57	113	882

**Table 12:** Class-wise comparison of accuracy between frameworks.

Framework	Normal	Mild	Moderate	Severe	PDR
**1**	99%	82%	86%	76%	69%
**2**	88%	90%	94%	97%	97%
**3**	84%	86%	94%	97%	98%

**Table 13:** Comparison of the proposed technique with existing methods.

Year	Author	Technique	Accuracy	Sensitivity
**2017**	[16]	AlexNet	97.93%	-
**2016**	[13]	CNN	75.00%	95.0
**2021**	[28]	Ensemble based Framework	70.00%	70.0%
**2021**	[29]	Ensemble based Framework	76.40%	-
**2020**	[30]	Ensemble based Framework	61.90	62.0%
**2019**	Proposed	Ensemble System	78.06%	78.0%
**2019**	Proposed	Ensemble System (Augmentation)	83.78%	82.24%

## Data Availability

Not applicable.
